# Co-insurance and health care utilization in Japanese patients with rheumatoid arthritis: a discontinuity regression approach

**DOI:** 10.1186/s12939-019-0920-7

**Published:** 2019-01-28

**Authors:** Jörg Mahlich, Rosarin Sruamsiri

**Affiliations:** 1 Düsseldorf Institute for Competition Economics (DICE), University of Düsseldorf, Universitätsstr. 1, 40225 Düsseldorf, Germany; 2 Health Economics and Outcomes Research, Janssen-Cilag, Neuss, Germany; 3 Center of Pharmaceutical Outcomes Research, Naresuan University, Phitsanulok, Thailand

## Abstract

**Background:**

Co-insurance rates in Japan decrease when patients turn 70 years of age. We aim to compare changes in medical demand for Japanese patients with rheumatoid arthritis (RA) at age 70 prior to 2014, when there was a reduction in co-insurance rates from 30 to 10%, with changes in medical demand at age 70 after 2014 when co-insurance rates decreased from 30% to only 20%.

**Methods:**

We used administrative data from large Japanese hospitals. We employed a discontinuity regression (RD) approach to control for unobserved endogeneity in the data.

**Results:**

We identified a total of 7343 patients with RA, 4905 (67%) turned age 70 before April, and found that a 20% decrease in co-insurance was associated with increased utilization of more expensive biologic RA drugs, more outpatient visits and higher total medical costs. However, a 10% decrease in co-insurance for patients who turned 70 after 2014 did not significantly change demand for medical services.

**Conclusions:**

For the younger cohort, we did not observe any changes in medical demand after a price decrease. We therefore conclude that the economic goal of cost sharing, namely a behavioural change towards lower health-care utilization, is not achieved in this particular cohort of chronic patients.

**Electronic supplementary material:**

The online version of this article (10.1186/s12939-019-0920-7) contains supplementary material, which is available to authorized users.

## Introduction

Medical insurance can increase the demand for medical care to a non-optimal level due to moral hazard [1]. In this context, moral hazard implies that patients do not consider the economic consequences of their behaviour, because under a free-care plan their marginal costs of health-care utilization is zero and only defined by their opportunity costs. To tackle this problem, some health-care systems have introduced cost-sharing schemes in health insurance such as co-payments or co-insurance. An argument often made is that co-payments reduce moral hazard for health-care utilization and eventually lead to a more efficient allocation of scarce resources. While the economic logic of this argument is compelling, there is limited empirical evidence to support this assertion. One exception is the famous RAND experiment of the 1970’s that randomized more than 5000 US citizens into different insurance schemes with different co-insurance rates [2]. Without co-insurance, the average total medical costs totalled 2170 USD, while the introduction of a 25% co-insurance reduced average costs by – 648 USD, and a 95% co-insurance further reduced costs by – 845 USD [3]. On average, the calculated price elasticity of demand for medical care was – 0.2, meaning that a price increase of 1% would reduce demand by 0.2% [4].

While the RAND study has been the only study in an experimental setting including randomization so far, there are a number of studies from several countries that evaluated the impact of co-payments and co-insurance using observational data. The results vary considerably across studies because of differences in methodology, institutional setting, and data aggregation, among other factors [5–10].

We identified three studies in Japan that analyzed the relationship between co-payments and patients’ utilization of health-care resources. All of these studies exploited a 20% reduction in the co-payment rate from 30 to 10% that was introduced for the majority of the Japanese population at age 70. For a general patient population Shigeoka (2014) and Fukushima et al. (2016) found that utilization of both inpatient and outpatient care services increased at age 70 due to the reduced co-insurance rate [11, 12]. However, the impact of reduced cost-sharing was not uniform across all medical services. A similar evaluation was performed on the impact of decreased cost-sharing on the use of dentures. Using data from the Japanese Study of Aging and Retirement (JSTAR), the utilization rate of dentures increased from approximately 50 to 63% around the threshold [13].

These studies, while informative, have some notable limitations. Firstly, because the co-payments for individuals older than age 70 were increased in 2014 from 10 to 20%, the conclusions may no longer be valid, warranting an additional analysis of the data. Secondly, the Fukushima study relied on the claims data set from JMDC (Japan Medical Data Vision), which includes insurance claims of employees and their dependents working for large Japanese corporations. Since the official retirement age in Japan is 65 years, very few people above this age remain in the database while those who do remain because they are still employed at the age of 70 are probably not representative of the Japanese population at large. Thirdly, the studies did not control for the impact of specific diseases. Of note, a financial aid system was introduced in Japan to help support patients with so-called intractable diseases (“nanbyou”), a group representing less than 0.1% of the population. These illnesses are associated with a high risk of disability and require labour-intensive care, which adds a heavy emotional and economic burden on family members. As of 2016, there were approximately 306 diseases classified as “nanbyou”, and medical care for patients with “nanbyou” diseases are heavily subsidized by the government. Those diseases have very low co-payment ceilings that often result in a marginal co-payment rates approaching zero. Given this special program, pooling patients across diseases can potentially introduce significant bias in the results. For this reason, in our investigation of the effects of co-payments on health-care utilization we have restricted our focus to only one disease entity, namely rheumatoid arthritis (RA), to exclude potential confounding factors. RA is a systemic autoimmune inflammatory disease, is mainly diagnosed in the elderly population who are particularly affected by changes in co-insurance rates when turning 70.

There are basically two medical treatment strategies available for RA: 1) Conventional disease-modifying anti-rheumatic drugs (cDMARDs) such as methotrexate (MTX), sulfasalazine (SSZ), leflunomide (LEF), or tacrolimus (TAC). All of these drugs are available as generics and therefore inexpensive. 2) Biologic DMARDs (bDMARDs) that have been available since the late 1990’s. bDMARDs have been shown to effectively delay and even prevent the clinical disease progression of RA [14]. Furthermore, treatment with biologic agents can improve productivity and help people retain productive employment [15]. There are several classes of bDMARDs available in Japan: tumor necrosis factor (TNF) inhibitors such as infliximab or golimumab, the interleukin-6 inhibitor tocilizumab, and the T-cell co-stimulation inhibitor abatacept [16, 17].

In this study, we evaluated the impact of lower co-insurance rates on drug utilization, utilization of inpatient and outpatient services and health-care costs for Japanese patients diagnosed with RA. We believe that RA is a good therapeutic area to study, because it is a chronic disease. For chronic diseases medical treatment cannot be avoided and co-payments might not be a suitable way to reduce costs.

## Methods

### Empirical approach

We exploited the current reduction in co-insurance in Japan from 30 to 20% for people at age 70. For individuals who turned 70 before April 1st 2014, co-insurance rates were reduced from 30 to 10%. High-income individuals earning more than 3.7 million Yen a year (32,137 USD using an exchange rate of 1 USD =115 JPY), a group that represents approximately around 9% of the Japanese population, were exempted from a co-insurance reduction. To limit the financial out-of-pocket burden, Japan imposed an income-dependent cap on co-payments (Table 1).

**Table 1:** Co-insurance rates and caps according to income and age

Subjects		Yearly income (disease)			Population		Co-payment	Maximum monthly burden on patients 2015		
		(in 10 thousands)						Up to 3 times a year		4 times or more
Lessthan the age of 70	High-income earner	1510	~		140	1.3%	30%	¥252,600	+ 1%	¥140,100
		1160	~	1510	190	1.8%				
		970	~	1160	360	3.4%		¥167,400	+ 1%	¥93,000
		770	~	970	640	6.1%				
	General	570	~	770	1450	13.7%		¥80,100	+ 1%	¥44,400
		370	~	570	2700	25.5%				
		310	~	370	1060	10.0%		¥57,600		
		100	~	310	3000	28.3%				
	Low-income earner		~	100	1050	9.9%		¥35,400		¥24,600
70–74	High-	570	~		20	3.1%	30%	¥44,400		
		370	~	570	40	5.5%				
	General	310	~	370	40	6.3%	20% (10% before April 1st 2014)	¥12,000		
		160	~	310	350	54.1%				
	Low-	80	~	160	140	21.1%		¥8000		
	income earner		~	80	60	9.8%				

Source: Bureau of Social Welfare and Health [18]

For people below age 70, the cap on co-insurance is 35,400 JPY (307 USD) for the population earning less than 1 million JPY (8700 USD). For the people with an income above 11.6 million JPY (100,900 USD) the ceiling is 252,600 JPY per month (2200 USD). For the latter group, there is an additional 1% co-payment rate that is not capped. If the ceiling is exceeded for more than 3 months in a year, the maximum amount is reduced to 24,600 JPY (213 USD) for the lowest, and 140,000 JPY (1217 USD) for the highest income groups. Of note, to benefit from the capped co-payment amount, patients must apply for a “certification card for the high-cost medical benefit system” with their health insurance company, though not all patients are aware of this system. Once a person turns 70, the upper limit of the co-payments is reduced drastically, ranging from 8000 (70 USD) to 80,000 JPY (696 USD) per month, depending on income and on whether the cost is related to inpatient or outpatient services [18]. Alternative plans have been put in place for people with specific, severe or “nanbyo” diseases. For those disease types the government provides partial financial assistance tosuch patients. Instead of the normal rate of 30% they are required to pay only 20% of their medical expenses out of pocket. Furthermore, there are different ceilings on co-payments. For patients who suffer from diseases on the “nanbyo” list, the maximum absolute amount of co-payment ranges between 2500 (21 USD) and 30,000 JPY (260 USD) per month, compared to the regular range of 35,400 (307 USD) to 252,600 JPY (2196 USD) per month (Additional file 1: Table S1) [19]. Because RA is not considered a “nanbyo” disease, RA patients are required to pay the full co-payment rates up to the higher ceilings.

### Data sources

We extracted commercially available hospital claims data from Medical Data Vision Co., Ltd., an administrative database for inpatients and outpatients that includes approximately 4,400,000 patients and represents approximately 3% of the total Japanese population. The age distribution in the database resembles that of the general population and is as follows: 0–14 years old: 13.5%; 15–64 years old: 52.4%; and 65 years and older: 34.1%. [20]. The data were obtained from hospital electronic information systems from 147 acute-phase hospitals throughout Japan and have been used in health-economic or epidemiological analyses of many different diseases such as schizophrenia [21], influenza [22], respiratory syncytial virus [23]. RA [24, 25], Cancer [26, 27], cardiovascular disease [28], and diabetes [29]. The evaluated hospitals provide 40,000 beds and are registered as Diagnosis Procedure Combination (DPC) hospitals. The DPC is a diagnosis-related group (DRG)-like flat fee system that was introduced in 2003 for big hospitals in Japan [30]. The time span of our analysis was from March 2009 to September 2015. Because data were de-identified by the database provider, no informed consent was necessary.

We identified patients with RA based on the International Classification of Diseases, 10th revision (ICD-10) [M05, M06.0, M06.2 - M06.9], who had at least one medication-based treatment for RA (e.g. DMARDs, bDMARDs or a painkiller). The index date was defined as the first visit at age 70. We included elderly patients between 68 and 71 years of age who were enrolled at least six months before and 12 months after turning 70. We used monthly data.

The following patient data were available: age, gender and co-morbidities. We calculated the Charlson Comorbidity Index (CCI) based on the reported comorbidities [31] and used previously described coding algorithms for defining comorbidities by Quan et al. [32]. The CCI was based on 17 comorbidities (myocardial infarction, congestive heart failure, peripheral vascular disease, cerebrovascular disease, dementia, chronic pulmonary disease, rheumatic disease including RA, peptic ulcer disease, mild liver disease, diabetes without chronic complication, diabetes with chronic complication, hemiplegia or paraplegia, renal disease, any malignancy including lymphoma and leukemia but except malignant skin neoplasms, moderate or severe liver disease, metastatic solid tumor, and AIDS/HIV) and gives a weight between 1 and 6 to each of these comorbidities. The higher the CCI, the higher the respective patient’s morbidity [31].

### Outcomes

We examined drug utilization, health-care utilization and health-care costs for each quarter. The following outcome variables were analyzed. *Drug utilization*: the percentage of patients receiving bDMARDs among treated patients with RA; the percentage of patients receiving bDMARDs among all patients with RA. *Health-care utilization per person month*: number of hospital admissions, number of re-hospitalizations, number of days in hospital, number of emergency room admissions. *Health-care cost per person month*: total cost (JPY per person month), outpatient (OPD) cost (JPY per person month), actual inpatient (IPD) cost (JPY per person month), DPC in-patient (IPD) cost (DPC -JPY per person month). All costs are converted to current prices using the customer price index [33].

We evaluated the proportion/percentage of patients who received bDMARDs, because biologics are an expensive treatment option compared with cDMARDs. In particular, we sought to determine if the reduction in cost sharing triggered an increase in the use of bDMARDs. We also report both total costs and costs for inpatient and outpatient medical services. The inpatient costs are further broken down into actual costs that accrue to the hospitals and DPC costs that are used for reimbursement by the health insurance companies. Hospitals make a profit when this flat fee exceeds their actual costs.

### Statistical analysis

To analyze the impact of cost sharing on the demand for medical services by patients with RA, we used a regression-discontinuity (RD) design [34]. The RD design uses discontinuities in the health insurance system to test causation by assigning subjects to either side of a cut-off value in order to determine an intervention’s effect [35]. This approach was widely used by health economics in several studies analyzing elderly patients in Japan [11, 12] and in the US [36, 37].

We conducted segmented regression analyses of the time-series data correcting for autocorrelated errors using the STATA arima command to estimate the effects of the co-payment change after turning 70 years of age [38]. This method allowed us to account for baseline levels and trends in each outcome measure while assessing changes in levels and trends following the co-payment change. Since we used aggregated time-series data at population level, our results were not affected by clustering [39].

We excluded the periods of 3 months before and 3 months after the patient reached age 70 as the roll-out period of our analysis. Fukushima et al. labelled this as a “donut hole” [12]. We chose this approach because people who know that cost sharing will be reduced at age 70 may postpone some medical treatments until after they turn 70. The “donut-hole” model removes such transitory responses and increases the robustness of the results. All analyses were performed in STATA V.14.0.

## Results

**Fig. 1:**
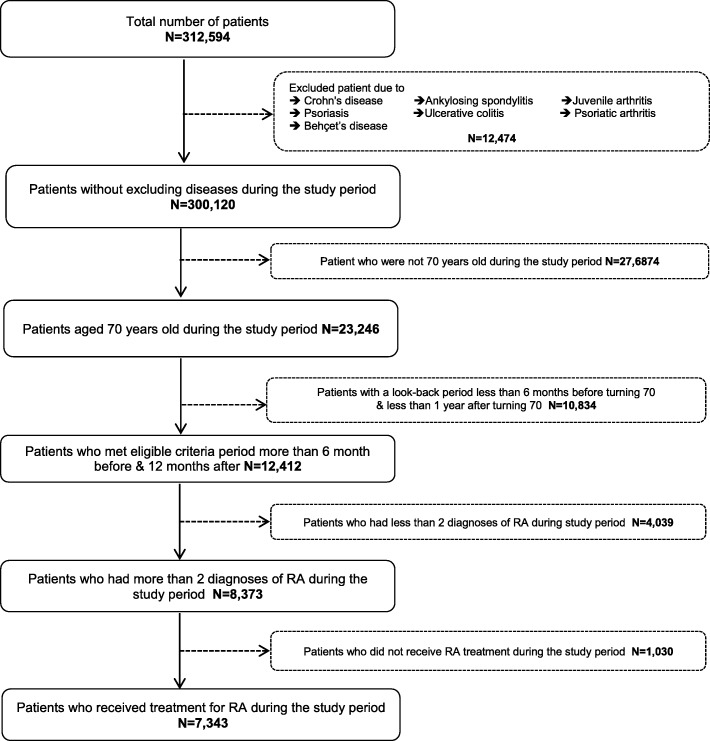
Patient selection

We included a total of 7343 patients with RA, 4905 (67%) turned age 70 before April 1st, 2014, and 2438 (33%) turned age 70 after this date (Fig. 1).

Table 2 reports the summary statistics of the patient population.

**Table 2:** Descriptive statistics of included patients

Characteristics	All N (%)	Pt. turning 70 before 2014	Pt. turning 70 after 2014	*P*-value
RA Patients	7343	4905 (67)	2438 (33)	
Age (mean + SD)	69.87 + 0.97	69.96 + 0.96	69.69 + 0.97	< 0.001
Gender				
Female	5874 (80)	3213 (79)	2661 (81)	0.065
Follow-up time (months) (mean + SD)	32.88 + 9.66	33.01 + 9.90	32.63 + 9.15	0.107
Comorbidity				
Pneumonia	2406 (33)	1628 (33)	778 (32)	0.271
Depression	352 (5)	221 (4)	131 (5)	0.101
COPD	1455 (20)	928 (19)	527 (21)	0.006
Liver disease	1263 (17)	823 (17)	440 (18)	0.175
Diabetes	775 (10)	459 (9)	316 (13)	< 0.001
Renal disease	341 (5)	203 (4)	138 (6)	0.004
CCI score (mean + SD)	2.12 + 1.94	2.00 + 1.84	2.35 + 2.10	< 0.001
≤ 2	5044 (69)	3450 (70)	1594 (65)	
3–5	1893 (26)	1228 (25)	665 (27)	
> 5	406 (5)	227 (5)	179 (7)	
Health-Care Utilization per person month (mean + SD)				
Outpatient visit	1.65 + 1.22	1.64 + 1.20	1.67 + 1.25	0.286
Inpatient visit	0.08 + 0.14	0.08 + 0.14	0.08 + 0.14	0.934
Length of stay	0.87 + 1.94	0.88 + 2.00	0.85 + 1.81	0.550
Number of re-hospitalizations	0.02 + 0.07	0.02 + 0.07	0.02 + 0.07	0.200
Number of emergency visits	0.01 + 0.03	0.01 + 0.03	0.01 + 0.02	0.028
Cost of treatment JYP per person month^a^ (mean + SD)				
Total health care cost	96,785 + 118,209	105,064 + 112,661	92,670 + 115,725	< 0.001
Out-patient cost	52,048 + 63,517	58,381 + 73,349	48,901 + 57,764	< 0.001
Hospitalization cost (actual)	44,736 + 95,002	46,683 + 93,367	43,768 + 95,799	0.216
Hospitalization cost (DPC)	38,869 + 85,578	39,369 + 80,196	38,607 + 88,137	0.710

*Pt*. patients, *SD* standard deviation, *RA* rheumatoid arthritis, *COPD* chronic obstructive pulmonary disease, *JPY* Japanese yen, *DPC* disease procedure combination

^a^ All monetary values were adjusted to 2016 values using the Japan CPI [33]

Consistent with international epidemiological studies [40] and Japanese data [41], the large majority (80%) of patients with RA patients in Japan were female. The mean follow-up period was 32.88 months. The most common co-morbidity was pneumonia, which occurred in 33% of the patients in our sample and is a frequent extra-articular manifestation of RA [42].

The mean CCI score was 2.12, and total health care costs were 96,785 JPY (841 USD) per month, with an average of 1.65 outpatient visits per month. The actual hospital costs were higher than the DPC costs. The results of the regression are reported in Table 3.

**Table 3:** Effect of co-insurance change in Japanese RA patients

Impact of co-payment change after turning 70	Intercept (95% CI)	Monthly baseline trend before (95% CI)	Level change immediately after (95% CI)	Monthly trend change after (95% CI)	Mean at age 69	Elasticity
1) Percentage of patients receiving bDMARDs among all RA patients						
Turned 70 before April 1st 2014	**14.77 (14.04–15.51)**	− 0.09 (− 0.17 - -0.02)	**3.13 (1.69–4.58)**	0.01 (− 0.07–0.10)	13.44	− 0.35
Turned 70 after April 1st 2014	**11.53 (10.36–12.71)**	0.05 (− 0.04–0.14)	0.50 (−1.13–2.11)	− 0.10 (− 0.21–0.01)	12.59	− 0.12
2) Percentage of patients receiving bDMARDs among treated RA patients						
Turned 70 before April 1st 2014	**47.13 (45.65–48.61)**	**−0.16 (− 0.29 - -0.02)**	**7.43 (4.79–10.07)**	−0.08 (− 0.26–0.10)	44.71	−0.25
Turned 70 after April 1st 2014	**36.29 (34.95–37.63)**	**0.31 (0.19–0.42)**	−0.53 (−2.92–1.84)	**−0.27 (− 0.43 - -0.11)**	42.17	0.04
3) Number of outpatient visits						
Turned 70 before April 1st 2014	**1.73 (1.71–1.74)**	**0.002 (0.0008–0.004)**	**0.09 (0.05–0.13)**	−0.003 (− 0.005–0.0001)	1.78	−0.08
Turned 70 after April 1st 2014	**1.85 (1.83–1.88)**	**− 0.005 (− 0.006 - -0.003)**	**−0.03 (− 0.07 - -0.004)**	0 (− 0.002–0.004)	1.80	0.05
4) Number of hospital admissions						
Turned 70 before April 1st 2014	**0.07 (0.07–0.08)**	0.001 (− 0.001–0.004)	0.003 (− 0.013–0.020)	−0.005 (− 0.012–0.003)	0.07	−0.06
Turned 70 after April 1st 2014	**0.06 (0.05–0.07)**	0.0006 (− 0.0002–0.001)	−0.005 (− 0.013–0.003)	0 (− 0.002 – 0.0002)	0.08	0.19
5) Number of days in hospital						
Turned 70 before April 1st 2014	**0.82 (0.76–0.89)**	0.002 (−0.002–0.007)	**−0.15 (− 0.27 - -0.02)**	0.005 (− 0.005–0.016)	0.88	0.26
Turned 70 after April 1st 2014	**0.65 (0.49–0.81)**	**0.01 (0–0.02)**	−0.01 (− 0.22–0.19)	**−0.02 (− 0.03 - -0.003)**	0.93	0.03
6) Number of re-hospitalizations						
Turned 70 before April 1st 2014	**0.02 (0.01–0.02)**	- 0.0001 (−0.0003–0)	**0.01 (0.004–0.017)**	0.0004 (0.0001–0.0007)	0.02	−0.75
Turned 70 after April 1st 2014	**0.02 (0.01–0.02)**	0 (−0.0002–0.0003)	0.001 (− 0.002–0.005)	0 (−0.001–0.00001)	0.02	−0.15
7) Number of ER visits						
Turned 70 before April 1st 2014	**0.01 (0.01–0.01)**	0 (−0.0002–0.0001)	−0.002 (− 0.005–0.001)	0 (−0.001–0.004)	0.01	0.30
Turned 70 after April 1st 2014	**0.006 (0.003–0.008)**	0 (0.0004–0.0004)	−0.002 (− 0.006–0.002)	0 (−0.0003–0.0001)	0.01	0.60
8) Total cost JPY per person month*						
Turned 70 before April 1st 2014	**96,219 (90,390 – 102,047)**	404 (−47–857)	**9939 (397–19,481)**	− 416 (− 1028–195)	107,196	−0.24
Turned 70 after April 1st 2014	**92,403 (88,335 – 96,471)**	102 (− 246–450)	− 3876 (− 11,099 – 3346)	458 (−151–1067)	95,423	0.12
9) OPD cost JPY per person month*						
Turned 70 before April 1st 2014	**58,170 (56,644 – 59,696)**	117 (−3–237)	**5667 (2212 – 9122)**	−48 (− 252–155)	61,129	−0.08
Turned 70 after April 1st 2014	**49,882 (48,900 – 50,863)**	**188 (108–268)**	− 1033 (− 3341 – 1274)	84 (− 57–227)	53,715	0.06
10) IPD cost (actual) JPY per person month*						
Turned 70 before April 1st 2014	**42,448 (38,932 – 45,963)**	−80 (− 376–215)	7534 (− 1904 – 16,972)	373 (− 287–1034)	46,067	−0.25
Turned 70 after April 1st 2014	**34,573 (28,538 – 40,608)**	184 (− 267–636)	−3014 (− 10,812 – 4783)	− 812 (− 1418 - -206)	41,707	0.22
11) IPD cost (DPC) JPY per person month*						
Turned 70 before April 1st 2014	**38,120 (30,575 – 45,665)**	285 (−254–826)	4522 (− 6336 – 15,381)	−387 (− 1092–316)	40,976	−0.17
Turned 70 after April 1st 2014	**32,903 (29,907 – 35,899)**	159 (− 77–396)	− 3032 (− 10,033 – 3968)	69 (− 516–654)	36,062	0.25

*bDMARDs* biologic disease-modifying anti-rheumatic drugs, *RA* rheumatoid arthritis, *CI* confidence interval, *DPC* disease procedure combination, *JPY* Japanese yen, *OPD* out-patient department, *IPD* in-patient department, *ER* emergency room

* All monetary values adjusted to 2016 values (Japan Consumer Price Index, 2017)

Bold numbers indicate significance at 5% level

For the cohort that turned 70 before April 1st 2014 and whose co-insurance rates decreased from 30 to 10%, there was a significant jump in bDMARD use, outpatient visits, re-hospitalizations, total costs and outpatient costs. However, we did not observe any significant change in the utilization of health-care resources for the patients who turned 70 after April 1st, 2014, and whose co-insurance rates were reduced to only 20%.

For the older cohort that saw a 20% decrease in their co-insurance rate, the total monthly costs increased by 9939 JPY (86 USD) in response to that reduction (Table 3). Of this increase in cost, 5667 JPY (49 USD) were due to outpatient-related services. For the younger cohort that saw only a 10%decrease in cost sharing, we even observed a slight drop in monthly health-care costs of - 3014 JPY (26 USD), although this change was not significant.

**Fig. 2:**
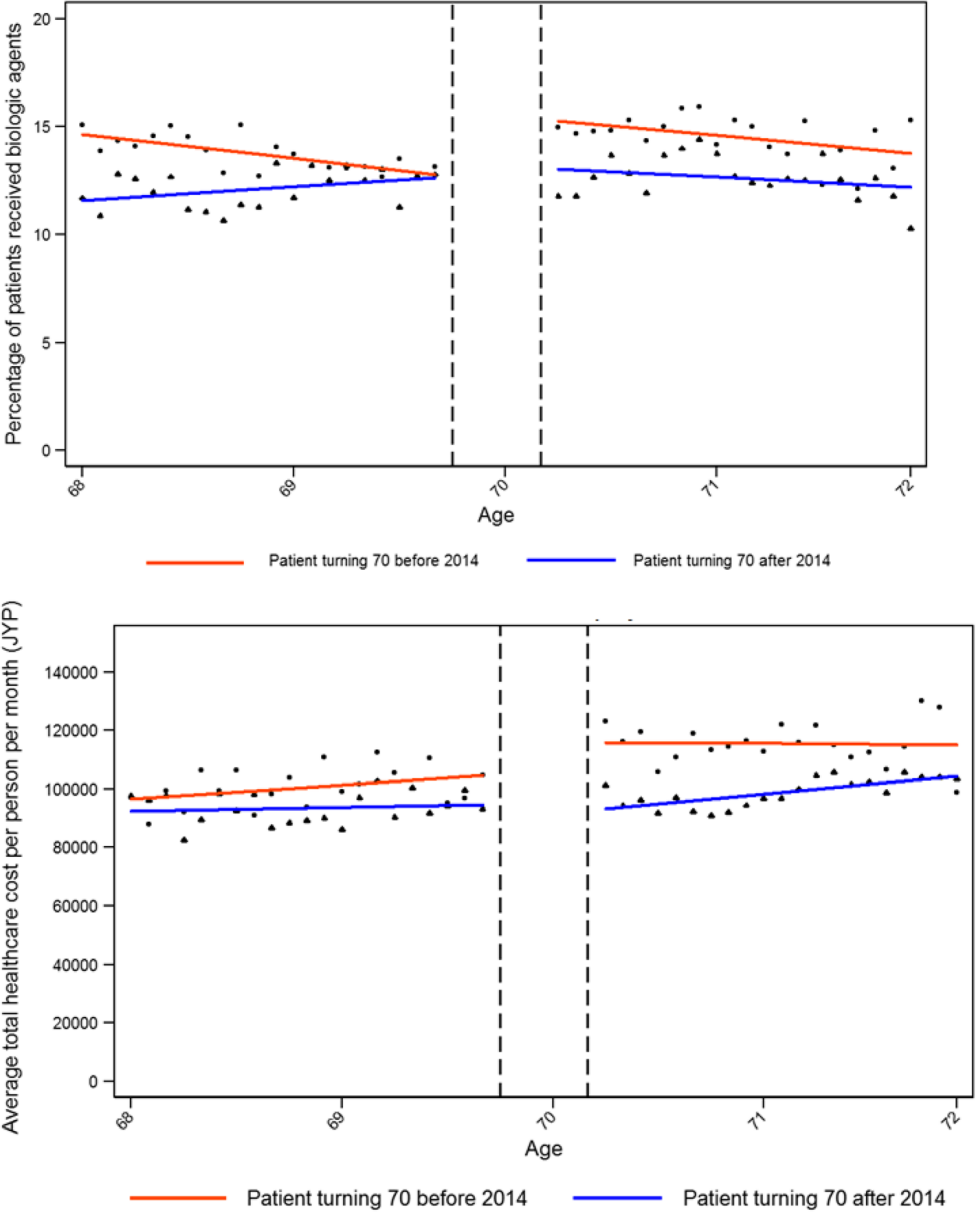
Share of patients receiving bDMARDs (top) and change in health-care cost (bottom)

To illustrate the results, Fig. 2 shows the proportion/percentage of patients receiving bDMARDS in the two cohorts. It nicely shows that the younger cohort’s demand for bDMARDs is unaffected by the reduction in co-payments.

## Discussion

We found that utilization of health-care resources by patients with RA increased when co-insurance rates dropped from 30 to 10%. These increases were reflected in the use of bDMARDs, outpatient visits, re-hospitalizations, total costs and outpatient costs. However, there was no increase in health resource utilization for the cohort of patients turning 70 after the health-care reform of 2014 that led to only a 10% reduction in co-insurance rates (30 to 20%). For this age group, the 10% (vs. 20%) reduction in co-insurance was not sufficient to trigger greater demand. Moreover, for this cohort, the estimated coefficients in most cases were actually positive, indicating a lower, though not significantly lower, resource utilization after a decrease in cost sharing. While this observation is at odds with conventional reasoning and with most empirical evidence [43]. Incidentally, a recent study in Israel had similarly unconventional results in that visits to physicians increased rather than decreased following the introduction of co-payments [44]. A potential explanation why elasticities are different between the two cohorts is that the demand function for drugs and medical services in our sample has a low price- elasticity when prices are low and vice versa. Those non-linearities were observed in a survey in Austria as well [45]. In that study, small co-payments had no effect on medical demand at all. Significant behavioural changes could be observed only when out-of-pocket payments of more than 100 Euro per doctor’s visit were introduced.

Demand elasticities for different types of medical services were heterogeneous, which is important from a policy perspective. To determine optimal cost sharing schemes, medical services that are more prone to moral hazard and whose demand is subject to higher price elasticity should be linked to higher cost-sharing in order to achieve a socially optimal allocation [46, 47]. In the US for example, health insurances such as Medicare apply different cost-sharing schemes to different medical services [48]. Compared to the US, responsiveness to price changes is lower in Japan, at least for the younger cohort. This finding may be explained by better and easier access to over-the-counter drugs and self-medication, which may replace physician visits and the use of prescription drugs [49]. Another possible explanation is the stereotypical image of health care in Japan, namely that doctors, who are honorifically referred to as “sensei” (teacher), tend to dictate treatment decisions that patients follow with little discussion [50]. These cultural differences may contribute to the greater ability of Japanese physicians to create a ‘supply-induced demand’ compared to their US counterparts.

Another explanation relates to our specific sample of patients with a chronic disease like RA. Earlier studies suggest that patients with chronic diseases visit their physician more often than healthier patients without chronic diseases while being less responsive to changes in co-payments or co-insurance rates [12].

As a result, co-payments may over-burden the chronically ill because of the necessity to seek treatment and the short supply of options available for reducing their use of health-care resources. This may also apply to the younger cohort of our sample. The discussion on equity effects of co-payments in Japan is currently ongoing. On the one hand, an ageing society is putting pressure on public healthcare budgets, but according to a survey of Japanese physicians, the majority of the respondents believe that the current level of co-payments deters patients from seeking adequate medical treatment. Within the last 6 months, 58% of the surveyed doctors have lost patients in follow-up due to the high financial burden of treatment. 52% reported that patients did not properly adhere to the treatment, and 62% even said that necessary medical tests could not be performed due to co-payments [51]. Physicians recommended adjusting the rate of co-payment to the type of disease and treatment. One potential solution is the introduction of policies aimed at reducing the use of medical services by patients with limited needs, providing access to affordable medical care for patients with chronic diseases by introducing disease-specific co-payments, and reduced rates for the treatment of chronic conditions.

Another major concern with patient cost-sharing is the so-called “offset effect”. This refers to the observation that raising the cost for physician visits or filling prescriptions through increases in cost-sharing can delay necessary care and increase emergency hospitalizations, if patients forgo necessary treatments. As a result, there are efficiency losses caused by under-treatment. For instance, the introduction of co-payments for medication has been associated with lower rates of drug treatment and lower adherence, which could lead to long-term economic costs due to under-treatment [52, 53]. Of note, the introduction of co-payments for prescription drugs in Canada resulted in more hospitalization events [54]. In addition, it was found that co-payments for the elderly in the US Medicare system resulted in a decrease in outpatient visits and increased hospital admission rates [55]. Hsu et al. reported that Medicare beneficiaries whose pharmacy benefits were subject to a cap had a 13% higher (non-elective) hospitalization rate and a 22% higher death rate than beneficiaries whose benefits were not capped [56]. Tamblyn et al. [57] reported that patient cost-sharing led to an increase in the rate of Emergency Department hospitalizations of the elderly of 14.2 per 10,000 patient-months in the 17 months after cost-sharing was instituted. Conversely, a recent Spanish analysis found that there was no reduction in the number of hospitalisations of the elderly after they were exempted from co-payments for prescription medicine [58].

Although the follow-up time in our study is too short to systematically analyze “offset-effects” in our population, there is less support for potential offset effects in Japan, because most hospital admission-related outcome variables did not change significantly, with the exception of re-hospitalisations within the older age cohort. However, the positive level of change indicates an increase in re-hospitalizations, when co-insurance rates decrease.

The major limitation of this study is the absence of data regarding individual income. We were therefore not able to evaluate marginal rates of co-insurance because co-payments are capped at levels that depend on income, and analysis rests on the assumption that income levels between the groups are similar. For this reason, the results provide only a rough estimate of actual elasticities and demand effects. Nevertheless, we believe that our results can serve as a basis for a discussion of policies to develop the optimal design for cost- sharing in Japan. Future research should look at other diseases as well to validate these findings.

## Conclusion

Our results suggest that in our sample of elderly patients with chronic rheumatoid arthritis, co-insurance rates do influence demand for medical care only in the group who turned 70 before April 2014 and whose co-insurance rates dropped from 30 to 10%. For the younger cohort that turned 70 after April 2014 and whose co-payment rates decreased only from 30 to 20% no changes in demand for medical care was observed. We therefore conclude that the current level of co-payment rates in this particular chronic disease does not accomplish the economic goal of cost sharing, namely inducing a behavioural change towards a lower health-care utilization. Co-payments that do not trigger behavioural changes just lead to a redistribution of the financial burden.

## Additional file

Additional file 1: **Table S1.** Special co-insurance for “intractable diseases” (nanbyou). (DOCX 14 kb)

## Abbreviations

bDMARDs: Biologic disease-modifying anti-rheumatic drugs
CCI: Charlson Comorbidity Index
cDMARDs: Conventional disease-modifying anti-rheumatic drugs
RA: Rheumatoid arthritis
RD: Regression discontinuity
