# Supplementary material for: Co-insurance and health care utilization in Japanese patients with rheumatoid arthritis: a discontinuity regression approach
Source: Int J Equity Health. 2019 Jan 28;18:22. doi: 10.1186/s12939-019-0920-7 (PMC6350300; doi:10.1186/s12939-019-0920-7)
Supplement: Supplementary file 1 — Table S1. Special co-insurance for “intractable diseases” (nanbyou). (DOCX 14 kb) [file 12939_2019_920_MOESM1_ESM.docx]

**Supplementary Table 1: Special co-insurance for “intractable diseases" (nanbyou)**

| **Income brackets (million JPY)** | **Monthly ceilings to 20%**  **co-payment (JPY)**  **First month** | **Monthly ceilings to 20%**  **co-payment (JPY)**  **Continuous treatment** |
| --- | --- | --- |
| Under public assistance | 0 | |
| < 0.8 | 2,500 | |
| 0.8 - 1.6 | 5,000 | |
| 1.6 - 3.7 | 10,000 | 5,000 |
| 3.7 - 8.1 | 20,000 | 10,000 |
| >8.1 | 30,000 | 20,000 |

Source:Ministry of Health [19]:
